# Removal of Cu(II) by Fixed-Bed Columns Using Alg-Ch and Alg-ChS Hydrogel Beads: Effect of Operating Conditions on the Mass Transfer Zone

**DOI:** 10.3390/polym12102345

**Published:** 2020-10-13

**Authors:** Ilse Paulina Verduzco-Navarro, Nely Rios-Donato, Carlos Federico Jasso-Gastinel, Álvaro de Jesús Martínez-Gómez, Eduardo Mendizábal

**Affiliations:** 1 Chemistry Department, CUCEI, University of Guadalajara, Blvd. Gral. Marcelino García Barragán 1421, Guadalajara, Jalisco 44430, Mexico; pau.verduzco@yahoo.com (I.P.V.-N.); nelyrios_2002@hotmail.com (N.R.-D.); 2 Chemical Engineering Department, CUCEI, University of Guadalajara, Blvd. Gral. Marcelino García Barragán 1421, Guadalajara, Jalisco 44430, Mexico; carlos.jasso@cucei.udg.mx (C.F.J.-G.); alvaro.martinez@cucei.udg.mx (Á.d.J.M.-G.)

**Keywords:** adsorption, chitosan, alginate, copper, fixed-bed

## Abstract

The removal of Cu(II) ions from aqueous solutions at a pH of 5.0 was carried out using fixed-bed columns packed with alginate-chitosan (Alg-Ch) or alginate-chitosan sulfate (Alg-ChS) hydrogel beads. The effect of the initial Cu(II) concentration, flow rate, pH, and height of the column on the amount of Cu removed by the column at the breakpoint and at the exhaustion point is reported. The pH of the solution at the column’s exit was initially higher than that at the entrance, and then decreased slowly. This pH increase was attributed to proton transfer from the aqueous solution to the amino and COO^−^ groups of the hydrogel. The effect of operating conditions on the mass transfer zone (MTZ) and the length of the unused bed (H_LUB_) is reported. At the lower flow rate and lower Cu(II) concentration used, the MTZ was completely developed and the column operated efficiently; by increasing column height, the MTZ has a better opportunity to develop fully. Experimental data were fitted to the fixed-bed Thomas model using a non-linear regression analysis and a good correspondence between experimental and Thomas model curves was observed.

## 1. Introduction

The availability of good quality water is essential for the sustenance of life. A large number of industries use considerable volumes of water and chemicals in their manufacturing processes, and as a result, a considerable amount of contaminated water is generated [1,2]. Among the heavy metals, copper is the most commonly discarded contaminant in wastewater [1]. Although copper is an essential nutrient at trace amounts, at higher levels, it is toxic to plants, algae, and humans [3,4]. There are several technologies for the elimination of metal ions from water such as chemical coagulation, biological treatment, Fenton, electrochemical oxidation, ozonation, ultrafiltration, electrocoagulation, and adsorption [5]. Several of the mentioned processes are not economically viable, and therefore, not suitable for their application [6]. Adsorption is a method used for its simple design and its easy applicability, because it implies low operating costs [7] and is very effective for the removal of low concentrations of the contaminant [8]. Although activated carbon is a quite effective universal adsorbent, its widespread use in water treatment is restricted due to its high cost; this is why adsorption using low cost adsorbents for water cleaning is an alternative and effective method [9]. Chitosan (Ch) is a low-cost bioadsorbent and has been used in batch systems for the removal of heavy metals due to the presence of amino and hydroxyl groups in its structure, which can interact with metals [10,11,12,13,14,15]. It has been reported that a derivative of Ch, a partially sulfated chitosan sulfate (ChS), is insoluble in a pH range of 2 to 12, and its amino and hydroxyl groups are responsible for the adsorption of metals [16,17]. The combined use of solutions of chitosan and sodium alginate in the treatment of wastewater containing heavy metal ions has been reported to be more effective for the removal of copper, cadmium, lead, and silver ions than when used alone [18]. Alginate is also a low-cost biopolymer, and because of its COO^–^ and OH groups, it has been used to remove metals [19,20].

For large-scale water treatment, continuous flow operations using packed columns are more convenient than batch systems, since they are simple to operate, have a greater removal efficiency, and can treat large volumes of water [21,22]. When operating an adsorption process in a batch system, the final concentration of the solution cannot be lower than the equilibrium concentration; in contrast, in a packed column, the output concentration can be zero or very small for a long time; therefore, a large volume of high-purity solution can be obtained [8]. To scale-up adsorption columns, it is necessary to run small-scale tests to obtain the length of the unused bed (mass transfer length or MTZ).

Although it has already been demonstrated that Ch and ChS are effective for the elimination of metal ions, since they are obtained as powder or flakes, they cannot be used to pack columns because they would cause a high drop in hydrodynamic pressure or clogging of the packed bed [23,24]. It has been reported that Ch and ChS dispersions in alginate hydrogel beads were used to remove copper ions from aqueous solutions at acidic pH in a batch process [16]. Cadmium (II) ions were removed from an aqueous solutions at pH = 5.0 using ChS dispersed in calcium alginate hydrogels using batch and continuous process [25].

Most of the reports on the use of fixed-bed columns for the adsorption of dyes or metal ions using low-cost biopolymer adsorbents such as chitosan composites are focused on the description and discussion of the effect of flow rate, pH, column height, and adsorbate concentration on the capacity of the columns at exhaustion [22]; however, it is necessary to know the adsorption capacity at both the breakthrough point and the exhaustion point to scale-up a column, since this two parameters allow the determination of the mass transfer zone (MTZ) and the MTZ length (H_LUB_) [8,26,27]. The ratio of unused bed length to total bed length (H_LUB_/H_T_ ratio) determines the efficiency of the process. When this ratio is less than 1.0, the MTZ is fully developed; therefore, the column is efficient [27]. When the H_LUB_/H_T_ ratio is small, most of the bed has been used at the breakthrough point, and a steep breakthrough curve is obtained [27].

When the MTZ is obtained from experimental data acquired at laboratory scale, and the H_LUB_/H_T_ ratio is less than 1.0, the column can be scaled-up [8,26].

As an aim, in this work, the removal of copper (II) ions from acidic aqueous solutions using fixed-bed columns packed with Ch and ChS dispersed in calcium alginate hydrogel beads is reported, looking for efficient conditions for adsorption. The effect of Cu(II) initial concentration, flow rate, pH, and column height on the shape of the breakthrough curves, and on the removal capacity at the breakthrough time, and at the exhausting point is presented. The effect of the experimental conditions on the MTZ value and the H_LUB_/H_T_ ratio is also reported. These parameters are required to determine whether the column is efficient and if column scaling is possible. Despite their importance, these parameters are rarely considered and reported in the literature. 

The fixed-bed Thomas model was applied here to the experimental data, and the obtained breakthrough curves were used to determine the MTZ length.

## 2. Materials and Methods 

### 2.1. Materials

**Figure 1:**
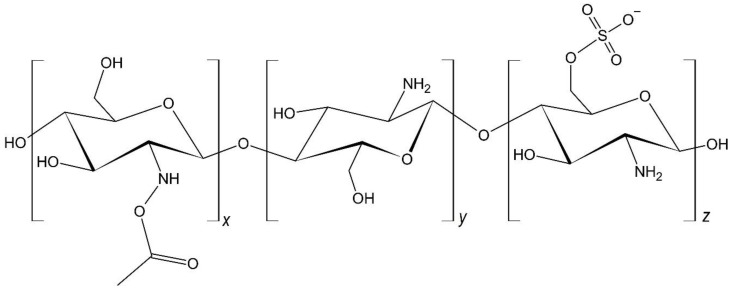
Structures of the ChS monomer units.

Food-grade chitosan with a 90% degree of deacetylation was purchased from América Alimentos (Zapopan, Mexico). Acetic acid (Fermont, Monterrey, Mexico), dimethylformamide (Fluka, Buchs, Germany), chlorosulfonic acid (Sigma-Aldrich, St. Louis, MO, USA), and methanol (Fermont, Monterrey, Mexico) were used as received. Chitosan sulfate (Figure 1) was synthesized from chitosan using the procedure previously reported [17]. Sodium alginate salt was obtained from Sigma Aldrich (Irvine, United Kingdom). Calcium chloride was obtained from Fermont (Monterrey, Mexico). Copper(II) ion solutions were prepared with copper sulfate pentahydrate salt (Fermont, Monterrey, Mexico) and bi-distilled water. The pH was adjusted with a 0.1 M HCl solution. 

### 2.2. Characterization of Chitosan and Chitosan Sulfate

Ch and ChS were characterized by Fourier transform infrared (FTIR) spectroscopy (Spectrum 100, Perkin Elemer, Seer Green, United Kingdom). The sulfur/nitrogen molar ratio (*S/N*) in the ChS was quantified in an elemental analyzer (TruSpec, LECO, St. Joseph, MI, United States), performing the analysis by duplicate. The percentage of protonable amino groups and the pK_a_’s of Ch and ChS were determined by potentiometric titration following the method reported by Ríos Donato et al. [25]; titration curves were obtained by titrating 0.2 g of Ch or ChS suspended in 10 mL of 0.1 M HCl with 0.1 M NaOH.

### 2.3. Preparation of Ch (Alg-Ch) and Alginate-ChS (Alg-ChS) Beads

First, the Ch and the ChS were pulverized in a mortar, and the portions that passed through a 200-sieve mesh (opening size of 75 micrometers) were used. A 1.5% (*w*/*w*) sodium alginate aqueous solution was prepared and the needed amount of Ch or ChS was added under stirring to obtain a dispersion. The mixture was poured into a pressure bottle, which was connected to an encapsulating equipment (Encapsulator B-390, Büchi, Flawil, Switzerland). To obtain the beads, the dispersion was passed through a nozzle of 1000 μm in diameter; a pressure of 250 mbar and a frequency of 600 Hz were applied. The beads were collected in a 0.1 M aqueous solution of CaCl_2_, leaving them in this solution during the time required for the calcium ions to diffuse into the pearls to crosslink the hydrogel. Next, the beads were washed and stored in bi-distilled water, in refrigeration.

### 2.4. Beads Characterization 

The morphology and surface area of the Alg-Ch and Alg-ChS beads were observed on a Hitachi TM 1000 scanning electron microscope operated at an acceleration voltage of 15.0 kV and an emission current of 48 mA. The average diameter of the beads was obtained by measuring 100 beads of each material using a digital electronic calibrator.

The concentration of Ch or ChS in the Alg-Ch and Alg-ChS hydrogels was obtained by gravimetry. The mass fraction of Ch or ChS (w_Ch or ChS_) was determined according to the following procedure; 1.0 g of beads was transferred to 15 mL tubes where 10 mL of a 0.1 M sodium citrate solution were added. The tubes were placed in a Thermoshaker (MCR, AccesoLab) at 100 RPM and a temperature of 25 °C until the calcium alginate matrix was dissolved. The solid was separated, dried, and weighed [28]. The mass of water (w_H2O_) and adsorbent (xerogel) was obtained by drying 1.0 g of beads in an oven at 50 °C until a constant weight was reached. The mass fraction of calcium alginate (w_Calcium alginate_) in the hydrogels was calculated using Equations (1)–(3).

(1) $$w_{\mathrm{Ch}\;\mathrm{or}\;\mathrm{ChS}}=\frac{\mathrm{mass}\;\mathrm{of}\;\mathrm{Ch}\;\mathrm{or}\;\mathrm{ChS}}{\mathrm{mass}\;\mathrm{of}\;\mathrm{hydrogel}}$$

(2) $$\;w_{H_{2}O}=\frac{\mathrm{mass}\;\mathrm{of}\;\mathrm{hydrogel}-\mathrm{mass}\;\mathrm{of}\;\mathrm{xerogel}}{\mathrm{mass}\;\mathrm{of}\;\mathrm{hydrogel}}\;$$

(3) $$\;w_{\mathrm{Calcium}\;\mathrm{alginate}}=1-w_{\mathrm{Ch}\;\mathrm{or}\;\mathrm{ChS}}-w_{H_{2}O}\;$$

### 2.5. Adsorption Equilibrium

Aqueous solutions with different concentrations of Cu(II) were prepared and aqueous solutions of 0.1 M HCl were used to adjust the pH to 5.0. The Alg-Ch or the Alg-ChS hydrogel beads were added to 10 mL of Cu(II) solution and the mixture was transferred to a 15 mL centrifuge vial. The tubes were placed in a Thermoshaker (MCR, AccesoLab) at 25 °C under continuous agitation (75 RPM) until equilibrium was reached (24 h). The solution was separated by decantation and the amount of Cu(II) remaining was determined with a Varian SpectrAA 220 flame atomic absorption system at a wavelength of 324.8 nm. The data obtained from the adsorption equilibrium tests were adjusted to the linearized Langmuir model [29] (Equation (4)):

(4) $$\frac{C_{e}}{q_{e}}=\frac{C_{e}}{q_{m}}+\frac{1}{q_{m}K_{L}}$$

where q_m_ is the maximum adsorption capacity (mg/g xerogel) and K_L_ is the Langmuir constant (L/mg). 

### 2.6. Fixed Bed Column Studies on Copper(II) Adsorption from Aqueous Solution

**Figure 2:**
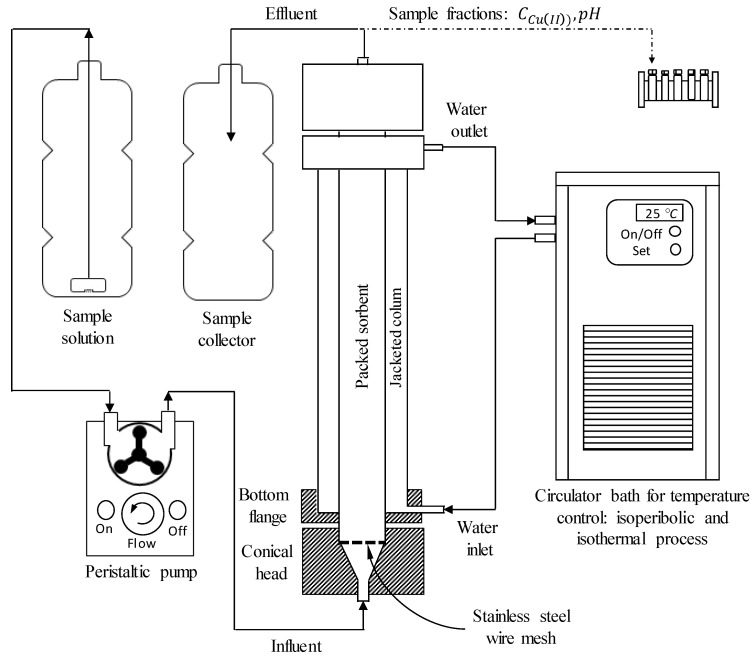
Experimental setup for column adsorption treatments.

The fixed-bed treatments were carried out at 25 °C. Glass columns were used with a bed height of 13 or 33 cm with an internal diameter of 1.8 cm; the columns were packed with 19 or 48 g of the hydrogel beads, respectively. The volume of the mobile phase in the spaces between the bead particles on the column, also referred to as the interstitial bed volume (IVB), of 13 cm height was 8.8 mL and 22.3 mL for that of the 33 cm height column. To feed the column with the copper(II) ion solution, a Masterflex 07557 peristaltic pump with Masterflex L/S 14 silicone hoses was used. A solution with a chosen predetermined concentration of copper(II) ions was fed to the bottom of the column at the desired flow rate regulated by the peristaltic pump (Figure 2). The flow direction was from the bottom to the top of the column, and samples were collected at different time intervals. The up-flow mode of operation was chosen to avoid channeling of the influent solution [30]. The copper ion concentration in the samples was measured using Varian SpectraAA 220 flame atomic absorption equipment at a wavelength of 324.8 nm.

### 2.7. Column Data Analysis

The determination of the adsorption capacity (exhaustion capacity) of the column was carried out using the profile of the advancing concentration in the fluid at the outlet of the bed. This profile is known as a breakthrough curve (or sigmoid curve), and is usually expressed as a dimensionless concentration ($C_{t}/C_{0}$) as a function of time [8]. $C_{t}$ is the copper concentration of the effluent at time t, while $C_{0}$ is the concentration of the feed solution. The value $C_{e}$ is usually considered as the point where the effluent concentration from the column is close to 100%, which is when the column’s exhaustion has been reached [26]. When the effluent concentration from the column reaches a maximum desired percentage of the influent concentration, it is considered that the breakthrough point has been attained ($t_{b}$) and is usually taken as 1 to 5% of $C_{0}$ [26]. When the effluent concentration is close to 100% of $C_{0}$, it is considered that the column is saturated. The total or stoichiometric capacity of the packed-bed column ($q_{e}$) can be obtained according to Equation (5) [8,26]:

(5) $$q_{e}=\;\frac{Q_{L}C_{0}}{m_{xerogel}}\left[\int_{0}^{t_{b}}\left(1-\frac{C_{t}}{C_{o}}\right)dt+\int_{t_{b}}^{t_{e}}\left(1-\frac{C_{t}}{C_{o}}\right)dt\right]=q_{b}+q_{MTZ}$$

where $Q_{L}$ is the volumetric flow rate; $t_{e}$ is the exhaustion time; and $m_{xerogel}$ is the mass of the xerogel. The integrals defined in Equation (5) are useful to scale-up the columns. The first integral is the time of the usable capacity of the adsorbate in the packed bed, and since $C_{t}$ in this time period is zero, or close to zero, the integral can be equated to $t_{b}$. The second integral corresponds to the time of the dimensionless concentration profile (sigmoidal curve) of the mass transfer zone (MTZ). MTZ is defined as the region of the fixed bed that is unsaturated [31]. The MTZ is constant as it travels through the column, if and only if, the length of the column is sufficient to contain a transfer zone in the steady state [32,33]. $q_{b}$ is the amount of adsorbate removed up to the breakthrough time, and $q_{MTZ}$ is the amount adsorbed in the MTZ.

### 2.8. Fixed Bed Model

The breakthrough curve (MTZ region) was obtained by fitting the experimental data of $C_{t}/C_{0}$ as a function of the number of IVBs that have passed through the column (NIVB), using the Thomas model [34]. The Thomas model is the most widespread model and is used to describe the behavior of the adsorption process in fixed bed columns [22]. This model assumes plug flow behavior in the bed and can be described by Equation (6):

(6) $$\frac{Ct}{C_{0}}=\frac{1}{1+exp\left(\frac{k_{Th}q_{t}m_{xerogel}}{Q_{L}}-k_{Th}C_{0}t\right)}$$

where $k_{Th}$ is the Thomas rate constant and $q_{t}$ is the total capacity of the packed-bed column. The parameters $k_{Th}$ and $q_{t}$ can be estimated by the Chi-square goodness-of-fit test using Minitab 18.

## 3. Results and Discussion

### 3.1. Characterization of Ch and ChS

#### 3.1.1. FTIR Spectroscopy

**Figure 3:**
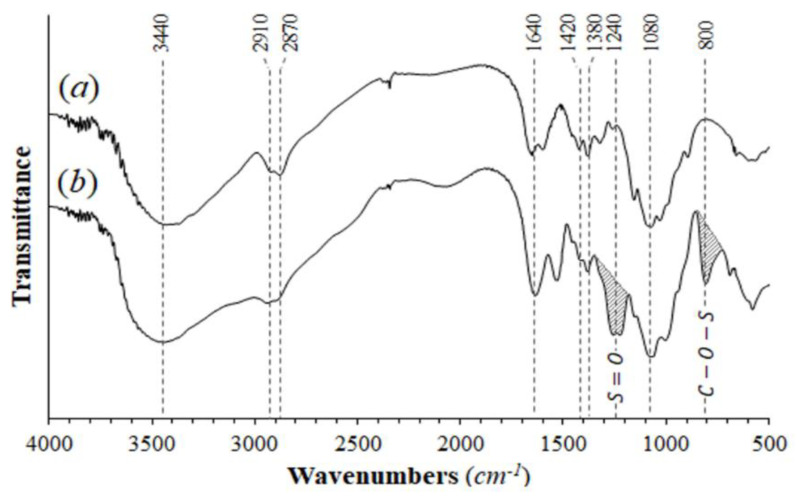
FTIR spectra: (**a**) chitosan and (**b**) chitosan sulfate.

Figure 3a shows a broad peak at around 3430 cm^-1^ due to the OH and amino groups, a peak of the carbonyl group at 1640 cm^−1^ and the pyranose ring signal at 1070 cm^−1^ [35]; all peaks were consistent with those reported for Ch [4,36]. The ChS spectrum (Figure 3b) showed, in addition to these peaks, stretch peaks of C–O–S (800 cm^−1^) and S=O (1250 cm^−1^), confirming that the ChS was obtained [37].

#### 3.1.2. Chemical Elementary Analysis of ChS

The elemental analysis of ChS indicated that the percentages of sulfur and nitrogen were 8.8% and 6.2%, respectively, which resulted in a *S/N* molar ratio of 0.63.

#### 3.1.3. Potentiometric Titration of Ch and ChS

Using the potentiometric titration data, two equivalence points were obtained for Ch ($V_{1}$ = 10.6 mL and $V_{2}$ = 20.5 mL titrating solution) and three equivalence points for ChS ($V_{1}\;$= 7.4 mL, $V_{2}$ = 11.1 mL and $V_{3}$ = 5.4 mL titrating solution), and with these data, a content of 83.2% and 81.3% of protonatable amino groups for Ch and ChS, respectively, was calculated. By using the Henderson–Hasselbalch equation [37], a pK_a_ value of 5.97 was obtained for Ch as well two pK_a_ values for ChS: the first equal to 5.67 and the second to 8.00.

### 3.2. Characterization of Hydrogel Beads

#### 3.2.1. Hydrogel Bead Composition

Through gravimetry, it was obtained that the Alg-Ch hydrogel beads contained 3.0% of alginate and 2.5% of Ch, and the Alg-ChS contained 3.0% of alginate and 0.7% of ChS. Higher concentrations of Ch and ChS could not be achieved in the hydrogels because the protonated amino groups of Ch and ChS interact with the carboxylate groups of the alginate, forming agglomerates, so that by increasing the amount of Ch or ChS, the agglomerates were larger and clogged the equipment’s nozzle.

#### 3.2.2. Average Diameter and Morphology of the Hydrogel Beads

**Figure 4:**
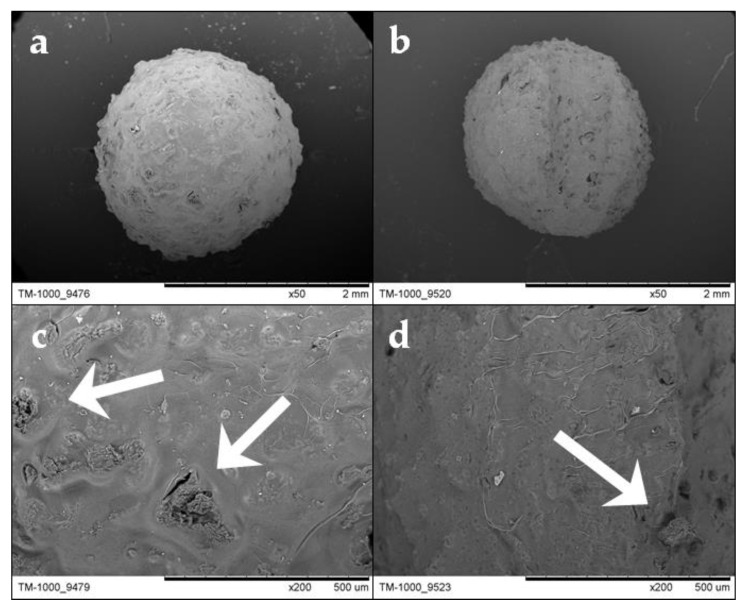
Image of an Alg-ChS bead (**a**) and an Alg-Ch bead (**b**) obtained with the scanning electron microscope (SEM) using 50× magnification. At 200× magnification, surfaces of the beads are shown, where particles of ChS (**c**) and Ch (**d**) are signaled with white arrows.

One hundred hydrogel beads were measured, resulting in the average diameter of the Alg-Ch beads being 2.07 ± 0.25 cm and that of the Alg-ChS being 2.09 ± 0.24 cm. The two types of beads (Alg-Ch and Alg-ChS) were almost spherical and had an irregular surface with pores (Figure 4a,b). The presence of pores enabled adsorbate ions to diffuse into the hydrogel beads during the adsorption process. ChS and Ch particles embedded in the alginate beads can be respectively observed in Figure 4c,d. There, the ChS particle signaled at the bottom center of Figure 4c seems to be well above 75 μm (mesh opening size), which supports the previously stated fact that ChS forms agglomerates when they are dispersed in alginate.

### 3.3. Adsorption Equilibrium

Table 1 shows the uptake capacity of the Alg-Ch and Alg-ChS xerogels (q_e_) and the Cu(II) concentration (C_e_) at equilibrium.

**Figure 5:**
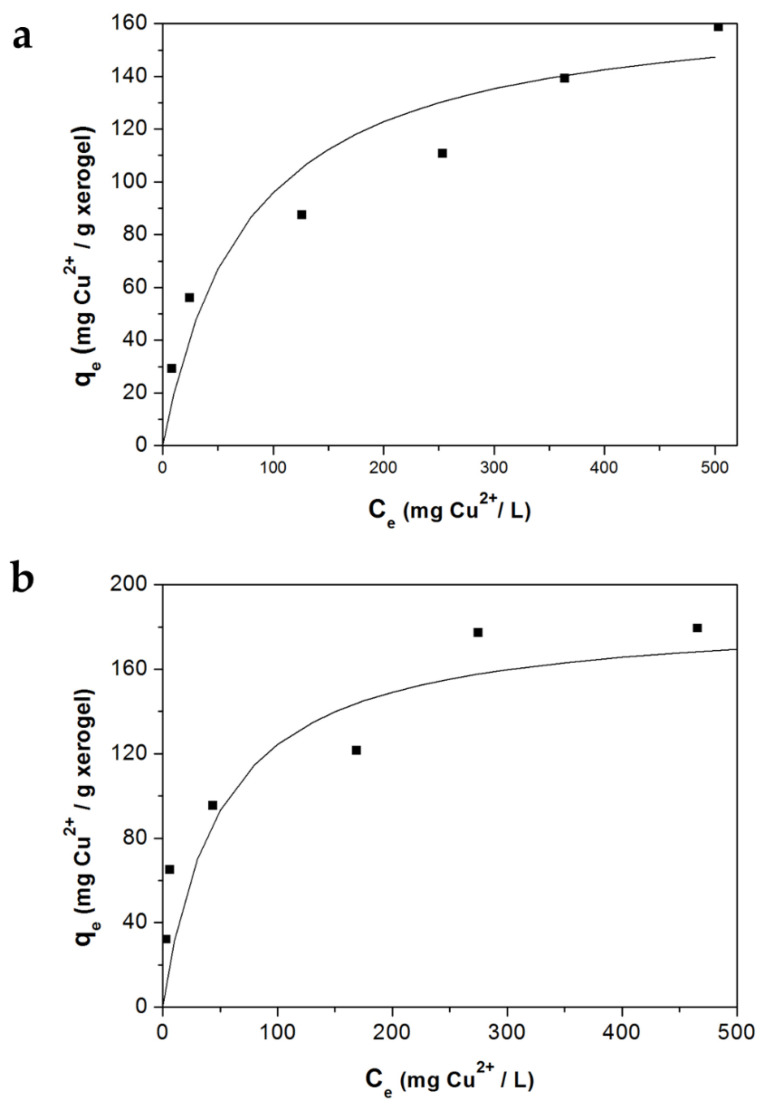
Cu(II) adsorption isotherms at the initial pH of 5.0 using (**a**) Alg-ChS hydrogel beads, (**b**) Alg-Ch beads. Experimental data (■) and Langmuir model (—).

By fitting the equilibrium data with the Langmuir model, a straight line was obtained, and the isotherm constants are reported in Table 2. Figure 5 shows that the Langmuir model closely fit the experimental data for the Alg-Ch and Alg-ChS xerogels.

### 3.4. Adsorption Studies in Fixed-Bed Columns

In Table 3, the following results are reported: the amount of Cu(II) removed by the Alg-Ch or Alg-ChS beads (mg of Cu(II)/g of xerogel) at the breakthrough point ($q_{b}$), at the end of the process ($q_{e}$), the length of bed used H_L_, the unused bed length H_LUB_, the time when the breakthrough occurs, and the number of interstitial bed volumes that have passed through the column (NIVB) at t_b_. When the Cu(II) concentration at the exit of the column was 5% of the concentration of the incoming solution, it was considered that the breakthrough point had been reached. The time at which the dimensionless concentration of the effluent reaches its maximum pre-defined permissible level ($t_{b}$) was estimated by analytical interpolation of the data of the breakthrough curve.

The breakthrough curve (MTZ) was obtained by fitting the experimental data to the Thomas model. The ratio ($q_{b}/q_{e}$) is the fraction of the capacity of the bed used up at the breakthrough point. Thus, for total length of the bed ($H_{T}$), $H_{L}$ is the length of the bed used up to the breakthrough point; in this region, the adsorbent particles are saturated, and $H_{L}$ can be obtained by the following equation [26,34,38]:

(7) $$H_{L}=\;\frac{H_{T}q_{b}}{q_{e}}$$

$\;H_{LUB}$ is the length of the MZT where the adsorbent particles are not saturated, and it is obtained by:

(8) $$H_{LUB}=\;H_{T}\left(1-\frac{q_{b}}{q_{e}}\right)$$

Although in common operation the flow in an adsorption column is stopped when the MTZ reaches the end of the column (the breakthrough point is reached) [32], it is necessary to continue adsorbate feeding until the exhaustion point to be able to determine $H_{LUB}$. This parameter is necessary for the columns’ scale-up [8,26,27,39]. The MZT length ($H_{LUB}$) is a critical parameter for the column design because it determines the length of the column used and it directly affects the feasibility and economics of the process [32]. When the MTZ length is very small (steep breakthrough curve), most of the bed has been used at the breakthrough point (H_L_ >> H_LUB_), so the column is highly efficient [8,34]. The MTZ length is constant as it travels through the column, if and only if, the length of the column is sufficient to contain a transfer zone in the steady state [32,33]. When the length of the column is insufficient, H_LUB_ (MTZ length) cannot be used for scaling up and the column has low efficiency.

**Figure 6:**
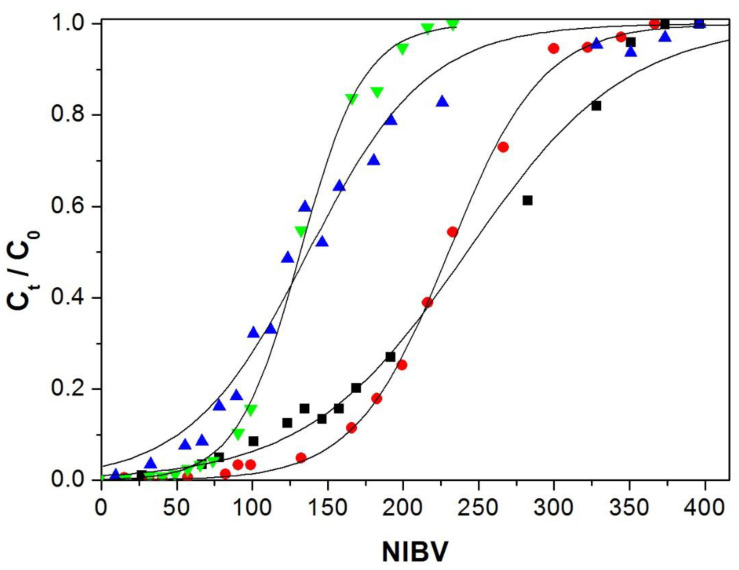
Breakthrough curves for Cu(II) adsorption, column height 13 cm and Cu(II) concentration 50 mg/L. Experimental Alg-ChS hydrogel: flow rate ● 50 mL/h, ■ 100 mL/h. Experimental Alg-Ch: flow rate ▼ 50 mL/h, ▲100 mL/h. Thomas model predictions (—).

**Figure 7:**
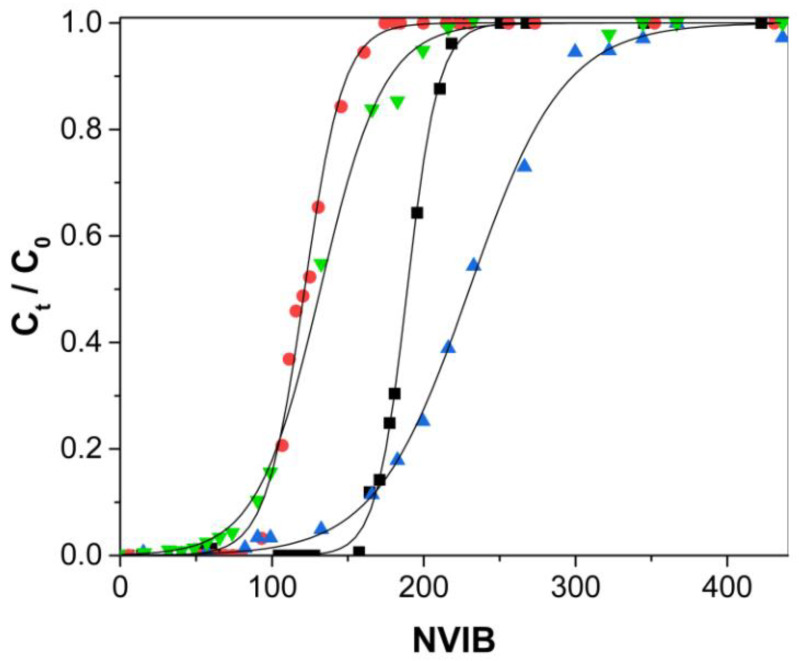
Breakthrough curves for Cu(II) adsorption, flow rate 50 mL/h, Cu(II) concentration 50 mg/L. Experimental Alg-Ch hydrogel, ▲ 13 cm and ■ 33 cm column height. Experimental breakthrough curves Alg-ChS hydrogel, ▼ 13 cm and ●33 cm. column height. Thomas model predictions (—).

**Figure 8:**
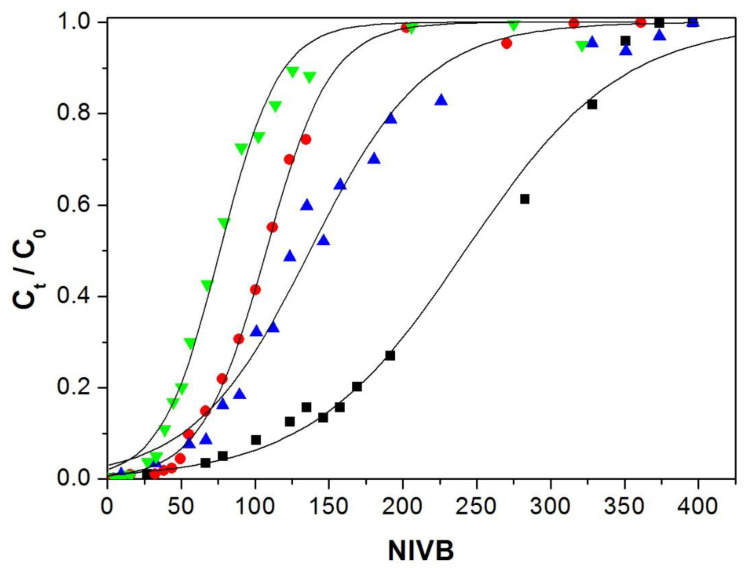
Breakthrough curves for Cu(II) adsorption, flow rate 100 mL/h, 13 cm height column. Experimental Alg-Ch: ■ 50 mg/L, ● 100 mg/L. Experimental Alg-ChS hydrogel: ▲ 50 mg/L, ▼ 100 mg/L. Thomas model predictions (—).

Figure 6, Figure 7 and Figure 8 show the breakthrough curves obtained by plotting C_t_/C_0_ against the number of interstitial bed volumes that have passed through the column (NIVB). These curves show the characteristic sigmoidal shape of the breakthrough curves obtained in adsorption using packed columns [8,22].

Table 3 shows that the highest bed capacity (*q_e_*) values were 101.6 and 99.0 mg Cu(II)/g xerogel for Alg-Ch and Alg-ChS, respectively. These values were higher than those reported for other low-cost adsorbents studied for the removal of Cu(II) at pH 5.0. A maximum bed capacity of 47.27 mg/g has been reported using kenaf fibers [40], 31.89 mg/g using magnetized sawdust [41], 58.23 mg/g for polyaniline-coated sawdust [42], and 16.52 mg/g when tethraethylenepentamine-modified sugarcane bagasse was used as the sorbent [43].

#### 3.4.1. Flow Effect

Aqueous solutions of 50 mg/L of Cu(II) at pH 5.0 were fed to columns of 13 cm in height, packed with 19 g of Alg-Ch or Alg-ChS hydrogel beads. Adsorption tests were performed using flow rates of 50 and 100 mL/h. Table 3 shows that by increasing the flow rate from 50 to 100 mL/h, when using Alg-Ch or Alg-ChS beads, q_b_ decreased noticeably and q_e_ was practically unmodified. When mass transfer resistances are negligible, the breakthrough curve will behave close to a step function, so by doubling the flow rate under a constant concentration, q_b_ should not be modified or be slightly different [8,27]. However, when there is solute dispersion, the breakthrough curves will be velocity-dependent and q_b_ will vary [8]. This variation can be attributed to the occurrence of various phenomena reported in column adsorption processes such as axial dispersion and external film resistance as well as intraparticle diffusion resistance [22,39]. Then, the decrease in q_b_ by increasing flow rate is due to a slow rate of adsorption because Cu(II) ions have to travel through the aqueous phase of the hydrogel to reach the active sites (amine and carboxylate groups), which makes the mass transfer slow. Although for any given time, at a higher flow rate, more Cu(II) passes through the fixed bed and the residence time of the solution is reduced, so the Cu(II) ions have less opportunity to interact with the adsorbent [44,45,46]. Table 3 also shows that at higher flow rate, the column becomes less efficient (H_L_ decreases and H_LUB_ increases) because the residence time of the solute in the column is not enough to reach equilibrium, resulting in a MTZ that is not fully established [32]. It has been reported that for Cu(II) adsorbed by chitosan immobilized in bentonite, the MTZ increased when the flow rate was augmented [47]. However, at the lower flow rate, H_LUB_/H_L_ was close to 1.0_,_ indicating that under these experimental conditions, the column was more efficient [27].

When using Alg-Ch beads, their values of q_b_ and q_e_ were larger than those obtained when Alg-ChS beads were used. This can be explained by intramolecular and intermolecular cross-linking reactions of the amino groups with the sulfate groups of ChS [48] and the carboxylate groups of the alginate [49], resulting in a decrease in the active sites of ChS and Alg.

Figure 6 and Table 3 show that when the flow rates were increased, smaller amounts of treated volumes were obtained at the breakthrough point (lower amount of Cu(II) removed), and a similar volume was needed to reach column exhaustion. It can also be observed that the breakthrough curves were steeper at the lower flow rate, indicating a closer approach to the ideal curve than at a higher flow rate. In the nickel(II) adsorption from aqueous solution by a treated polyurethane foam, breakthrough curves plotted as a function of time were reported to be steeper when the flow rate was increased; however, it resulted in lower efficiency [50]. In the columns packed with Alg-ChS, the breakthrough and saturation curves appeared at a lower NIVB than those obtained with the columns containing Alg-Ch because they had less adsorption capacity. In the removal of Cu(II) using a fixed-bed column, breakthrough curves plotted as a function of time were steeper at higher flow rates, but q_b_ was smaller [47]. When breakthrough curves are plotted as a function of time, they become steeper when increasing the flow rate because the breakthrough and exhaustion time occurs earlier [47]. This could give the erroneous idea that the higher the flow rate, the closer to the ideal adsorption curve.

#### 3.4.2. Effect of Bed Height

The bed height effect was determined by the comparison of the Cu(II) removal capacity of the 13 cm high column with that of the 33 cm high column. Aqueous Cu(II) solutions at a pH of 5.0 and a concentration of 50 mg/L were fed to the columns at a flow rate of 50 mL/h. Table 3 shows that, regardless of the adsorbent type or initial concentration, higher q_b_ was obtained when using the larger column, and that q_e_ is practically unmodified. The higher q_b_ can be explained by the larger number of active adsorbent sites available to interact with the solute before the solution leaves the column [51]. This is in accordance with literature reports that larger q_b_ is obtained by increasing the bed height [52,53,54].

When the larger columns were used, values of the H_LUB_/H_L_ ratios were less than 1.0, indicating that a longer column improves the process’s efficiency because there is more time for the MTZ to approach full development. The MTZ is fully developed when the values of the H_LUB_/H_L_ ratios are below 1.0 [32].

Figure 7 and Table 3 show that when the length of the column was increased (higher amount of adsorbent), more volume was passed through the column before the breakthrough point was reached, and steeper curves were obtained, indicating greater efficiency. It has been reported that when plotting the breakthrough curves as a function of time, by increasing the length of the column, the curves become less steep [23,47,52,53,55] as the MTZ requires more time to reach the end of the column, which increases the breakthrough times [46].

#### 3.4.3. Effect of Copper(II) Ion Concentration

Solutions of Cu(II) ions with a pH of 5.0 were fed at a flow rate of 100 mL/h through the column of 13 cm in height packed with Alg-Ch or Alg-ChS hydrogel beads. The Cu(II) concentrations fed to the column were 50 and 100 mg/L. Table 3 indicates that, independently of the type of adsorbent used, when a higher concentration of Cu(II) was employed, q_b_ increased slightly due to the larger mass transfer driving force (larger concentration gradient) [56,57], but q_e_ decreased slightly and MTZ length increased with an adsorbate concentration increment. A similar result was reported for the removal of copper using chitosan immobilized in bentonite [47].The ratio H_LUB_/H_L_ in all cases was much larger than 1.0, indicating that the MTZ had not been fully developed, so the columns did not efficiently remove Cu(II) under this high flow. The decrease in q_e_ can be explained because at a high flow rate, the time for the Cu(II) to contact the adsorbent is not sufficient for the system to reach equilibrium before the solution leaves the column [58].

Figure 8 shows that by increasing Cu(II) concentration, the breakthrough curves move to fewer NIVB and are steeper, resulting in a smaller amount of volume passing through the column at the breakpoint and at exhaustion. It has been reported that steeper breakthrough curves are obtained, and they shift to shorter times (lower treated volume) when larger adsorbate concentration is used because the time to reach the breakthrough point and the column exhaustion is reduced [47,59]. When Alg-ChS beads are used, the breakthrough curves move to smaller NVIB values compared to when Alg-ChS beads are used.

#### 3.4.4. pH Effect

**Figure 9:**
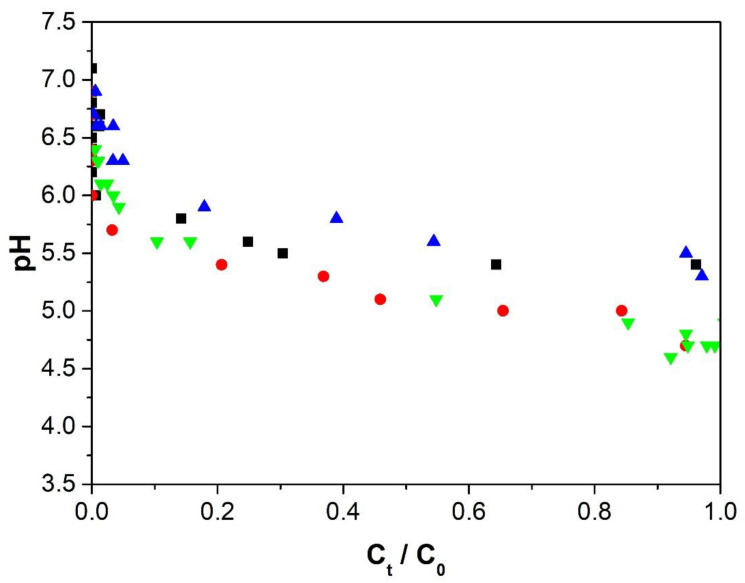
pH of the effluent solution using a flow rate of 50 mL/h, initial concentration of 50 mg/L, and pH of 5.0. Alg-Ch: ■ 33 cm, ▲ 13 cm column height;. Alg-ChS: ● 33 cm, ▼ 13 cm column height.

Figure 9 shows the exiting aqueous solution’s pH as a function of C_t_/C_0_, and that initially, the solution leaves the column at a much higher pH than that of the incoming solution (pH = 5.0). The increase in pH is due to the transfer of protons from the solution to the amino and carboxylate groups of the adsorbate. Therefore, there is competition between the protons of the aqueous medium and Cu(II) ions to interact with the amino and carboxylate groups during the adsorption process. For the alginate, the interaction of calcium alginate carboxylate moieties with either protons or Cu(II) ions is controlled by ion exchange and coordination reactions [60]. For Ch and ChS, Equations (9) and (10) depict the interaction between the unpaired electrons of the nitrogen atom of the amine group with Cu(II) ions and with protons [10], respectively.

(9) $$Cu^{2+}+NH_{2}-R\to Cu^{2+}NH_{2}-R$$

(10) $$\;H^{+}+NH_{2}-R\to NH_{3}{}^{+}-R\;$$

Figure 9 also shows that the pH decreases as the solution passes through the column because the number of amino and carboxylate groups available to interact with the protons declines. This adsorption process is complex because in addition to the mass transfer resistance, axial dispersion and equilibrium phenomena [61], the pH gradient inside the column is being modified during the adsorption process. During the removal of heavy metal ions using cellulose/chitin beads, it has been reported that an initial high increase in the pH of the output solution is followed by a slow decrease [62].

As Ch is soluble at an acidic pH, a qualitative test was performed to determine if Ch dissolved when the solution passed through the column. Samples were taken from the effluent, and an aqueous solution of 1.0 M NaOH was added to increase the pH to approximately 12.0. If Ch was contained in the effluent, it would precipitate. The output solution at the point of rupture (pH 6.3–6.6) showed no precipitation. However, at the end of the process, the pH of the effluent was close to 5.0, and in the qualitative tests, it was observed that some Ch had precipitated. As the partially sulfated ChS was not soluble at acidic pH, no precipitate was observed in the effluent during the whole adsorption process.

### 3.5. Modeling of the Behavior of the Fixed-Bed Column

Experimental data were fitted to the fixed-bed Thomas model (Equation (6)) using non-linear regression analysis at a 95% significance level. The corresponding model parameters are shown in Table 4. Thomas model breakthrough curves for the treatments are shown in Figure 6, Figure 7 and Figure 8, where good correspondence between the experimental and Thomas model curves was observed. This concordance was consistent with the small standard deviation (SD) values obtained, which varied from 0.018 to 0.045. The SD represents the distance that separates the experimental data values from the values adjusted by the Thomas model.

## 4. Conclusions

For the experimental conditions used in this work in the fixed-bed columns, the full development of the MTZ could be obtained by using the smallest flow rate and lowest Cu(II) concentration (50 mL/h and 50 mg/L). When the height of the column was increased under the same operating conditions, q_b_ increased significantly, and the H_LUB_/H_T_ ratio decreased; this indicates that a greater percentage of the column was used to adsorb Cu(II).

To determine the conditions for column scale-up, it is recommended to perform experimental runs by varying the flow rate, initial solute concentration, and column height to determine the operating conditions that enable the column to be efficient.

If chitosan or one of its derivatives is used as an adsorbent, it is necessary to measure the pH of the solution leaving the column to keep observing its adsorption capability and to have a better understanding and control of the adsorption process.

Despite the complexity of this adsorption process, there was good correspondence between the experimental data and Thomas’ model, which allowed the estimation of the MTZ.

The Alg-Ch and Alg-ChS beads showed a higher capacity for Cu(II) removal than other biosorbents. When using Alg-Ch beads, the q_b_ and q_e_ values were greater than those obtained when Alg-ChS beads were used. However, Alg-ChS does not undergo dissolution at low pH values (pH < 6), as Alg-Ch does.

## Figures and Tables

**Table 1:** Cu(II) adsorption onto Alg-Ch and Alg-ChS hydrogel beads at equilibrium at 25 °C and initial pH = 5.0.

Alg-Ch		Alg-ChS	
C_e_ (mg/L)	q_e_ (mg/g xerogel)	C_e_ (mg/L)	q_e_ (mg/g xerogel)
2.76	32.1	8.5	29.2
5.99	65.1	24.4	56.1
43.6	95.4	126.1	87.5
168.7	121.5	253.5	110.6
274.6	177.3	364.1	139.2
465.4	179.3	503.2	158.6

**Table 2:** Isotherm constants of the Langmuir model for adsorption of Cu(II).

Alg-Ch			Alg-ChS		
q_m_ (mg/g)	K_L_ (L/mg)	R^2^	q_m_ (mg/g)	K_L_ (L/mg)	R^2^
186	0.0201	0.930	170	0.013	0.960

**Table 3:** Results obtained in the Cu(II) adsorption at pH 5.0 using fixed-bed columns filled with Alg-Ch or Alg-ChS hydrogel beads.

Hydrogel Type	C_0_ mg/L	Flow rate (mL/h)	H_T_ cm	H_L_ (cm)	H_LUB_ (cm)	t_b_ (h)	t_e_ (h)	NIVB ^1^ at t_b_	q_b_ (mg Cu^2+^/g xerogel)	q_e_ (mg Cu^2+^/g^−1^ xerogel)
Alg-Ch	50	50	13.0	6.4	6.6	20.2	62.6	111.8	48.3	99.5
	50	100	13.0	4.0	9.0	6.6	32.0	73.9	28.9	94.6
	100	100	13.0	4.4	8.6	3.5	25.3	38.7	29.8	88.0
	50	50	33.0	27.6	5.4	71.9	100.2	157.3	85.1	101.6
Alg-ChS	50	50	13.0	6.8	6.2	12.1	38.2	70.6	45.2	87.0
	50	100	13.0	3.7	9.3	3.9	36.1	43.8	25.3	89.6
	100	100	13.0	3.6	9.4	2.0	25.6	22.4	26	94.9
	50	50	33.0	27.3	7.7	42.0	75.0	93.6	75.4	99.0

^1^ IVB = 8.8 mL for the 13 cm height column; IVB = 22.3 mL for the 33 cm height column.

**Table 4:** Thomas model parameters obtained using nonlinear regression analysis.

Hydrogel Type	C_0_	Q_L_	H_T_	q_Th_	k_Th_ × 10^3^	SD
	$\;\left(\frac{mg\;Cu^{+2}}{L}\right)\;$	$\;\left(\frac{cm^{3}}{h}\right)\;$	(cm)	$\;\left(\frac{mg\;Cu^{+2}}{g\;xerogel}\right)\;$	$\;\left(\frac{L}{h\;mg\;Cu^{+2}}\right)\;$	Dimensionless
Alg-Ch	50	50	13	99.6	3.30	0.018
	50	100	13	96.5	4.07	0.042
	100	100	13	81.1	5.67	0.033
	50	50	33	101.5	2.94	0.040
Alg-ChS	50	50	13	86.5	4.50	0.039
	50	100	13	79.9	6.21	0.044
	100	100	13	86.0	6.20	0.036
	50	50	33	97.3	2.66	0.045
